# Designing age-friendly interfaces and wearable–environment integrations to enhance medication adherence: a discrete choice experiment among older adults

**DOI:** 10.3389/fmed.2026.1749507

**Published:** 2026-03-11

**Authors:** Jialin Qin, Zeyu Wu, Hao Zhang, Xiaopan Qi

**Affiliations:** 1 Faculty of Humanities and Arts, Macau University of Science and Technology, Taipa, Macao SAR, China; 2 Shenzhen Library, Shenzhen, China

**Keywords:** discrete choice experiment, environmental adaptation, interface design, medication adherence, older adults, wearable integration

## Abstract

**Introduction:**

With population aging, the burden of chronic diseases is increasing, placing substantial pressure on individuals and health systems. Poor medication adherence remains a major barrier to effective chronic disease management and the efficient use of healthcare resources. Existing interventions rely largely on education or single-modality reminders, and systematic empirical research on interface design, wearable integration, and environmental adaptation remains limited.

**Methods:**

To address this gap, this study employed an extended discrete choice experiment (DCE) framework to quantify the preference structures of older users regarding reminder modality, confirmation method, font and layout, wearable integration, and environmental adaptation strategies. A total of 203 valid responses were collected, and overall trends and group differences were examined using a mixed logit model and latent class analysis.

**Results:**

The mixed logit model showed that older adults exhibited positive utility estimates for several interface attributes, including multimodal reminders, adaptive font and layout, and single-tap confirmation. They also preferred coordinated smartphone–smartwatch use under the wearable integration attribute. For environmental factors, both context-adaptive and biophilic themes were associated with positive utility estimates. These themes were implemented through interface-level visual cues, including natural color palettes, background imagery, and context-responsive visual adjustments, suggesting that alignment between interface cues and environmental elements can enhance adherence motivation. The latent class analysis further identified two user groups. The Efficiency–Context group (84.2%) preferred simple, efficient, and low-burden interactions, while the Cue–Wearable group (15.8%) valued multimodal prompting and device coordination but showed limited responsiveness to layout or environmental themes.

**Discussion:**

Overall, the findings suggest that optimizing interface and environmental elements can support medication adherence among older adults and provide quantitative design evidence for age-friendly digital health systems.

## 1 Introduction

Population aging is one of the most significant demographic shifts of the 21st century. By 2050, the global population aged 60 years and older is projected to reach 2.1 billion (1). This demographic trend is expected to intensify the burden of chronic diseases (2). At the same time, poor medication adherence among older adults has emerged as a critical global public health concern. Evidence shows that non-adherence among individuals aged 50 years and above is associated with higher hospitalization and mortality rates, whereas adherence is linked to an approximately 21.0% reduction in mortality risk (3).

Regional studies further demonstrate the scale of this issue. In the Middle East, non-adherence among home-dwelling older adults with chronic conditions reaches 67.9% (4). In China, medication adherence among older adults with hypertension remains low (5), and poor adherence is closely linked to limited health education, weaker self-management ability, and rural residence (6). A study of older stroke survivors reported that about 61.4% were non-adherent, with risks increasing alongside higher medication burden and lower health literacy, contributing to further medical strain (7). Taken together, medication non-adherence is widespread across regions and healthcare systems and represents a major barrier to effective chronic disease management in the context of global population aging.

In recent years, internet-based medical platforms have been widely applied in older adults’ health management (8, 9). Existing interventions aimed at improving medication adherence largely rely on reminder- or education-based approaches, including telephone follow-ups, SMS or WeChat notifications, and health education campaigns (10–12). Although combined interventions integrating education and reminders have been examined extensively, their effectiveness in improving medication-taking behavior among older adults remains limited (13, 14). Educational programs and remote health consultations have also yielded modest outcomes among older adults with chronic conditions (15). Evidence further suggests that mobile applications may improve adherence among individuals with chronic diseases, although the overall quality of evidence is low, intervention periods are short, and systematic definitions of ideal application functions remain lacking (16–18).

Research on mobile health (mHealth) applications for older adults has examined accessibility features such as large font size, high contrast, and voice interaction, yet most studies focus on single interface components (19, 20). Prior work emphasizes universal design principles intended to reduce cognitive and operational burden and improve usability, although most interventions remain centered on reminders and educational strategies (19). Methodological heterogeneity also remains substantial across studies, with diverse variable configurations and the absence of standardized intervention protocols and measurement indicators limiting comparability (21).

Therefore, this study addresses the lack of integrated empirical evidence by examining how multiple interface, wearable, and environmental design attributes jointly shape older adults’ medication-adherence choices within a unified experimental framework. This study focuses on answering the following two questions:

RQ1: How are older adults’ preferences reflected in their choices of reminder modality, confirmation burden, font and layout, wearable integration, and environmental adaptation in medication-adherence scenarios?

RQ2: What latent preference patterns emerge among older adults, and what significant differences exist across user groups in their selections of interface design attributes?

To address these questions, this study targeted older adults aged 60 years and above who have chronic conditions and can use smartphones independently. An extended discrete choice experiment (DCE) framework was employed to construct experimental combinations across five attributes: reminder modality, confirmation method, font and layout, wearable integration, and environmental adaptation. Overall preferences were estimated using a mixed logit model (MLM), followed by latent class analysis (LCA) to identify heterogeneity across user groups. The findings aim to provide quantitative design evidence for age-friendly digital health systems, enrich the interface-design perspective within medication adherence research, and offer practical guidance for improving wearable health products and mobile healthcare applications.

## 2 Related works

### 2.1 Interface and interaction design factors

In recent years, “age-friendly” interface design has become an important topic in research on mobile health applications (22–24). Prior studies have indicated that interfaces for older adults should remain simple and flexible, aiming to reduce cognitive and operational load. They recommend avoiding handwritten or decorative fonts, instead adopting serif or sans-serif typefaces, and ensuring adequate font size and high contrast to improve readability and visual comfort (25, 26). A review further reported that older users prefer larger font sizes, although there is a threshold beyond which excessively large text may reduce readability (27). Additional research on “adaptive interfaces” suggests that interfaces can dynamically adjust font and layout, as well as notification styles, based on the user’s status to reduce interaction burden for older adults (28).

In terms of interaction methods, studies have shown that medication management applications based on barcode or QR code recognition can convert medication information into reminder instructions, thereby significantly improving safety for older adults who take multiple medications (29, 30).

Although the above studies offer valuable insights, most examine isolated interface elements or a single confirmation approach. To support more design-relevant comparisons, this study incorporates key interface and interaction attributes into an experimental choice framework to estimate older adults’ preferences under medication-management scenarios.

### 2.2 Wearable devices and multimodal reminders

At present, multimodal reminders and wearable devices have been widely used to support health-related behaviors and are increasingly applied in medication management. Systematic reviews have shown that wearable devices can provide continuous status monitoring and chronic disease management prompts in remote settings (31). However, most studies remain focused on feasibility, and empirical evidence linking cross-modal reminders to actual behavioral outcomes remains relatively limited.

Regarding direct evidence on the role of wearable devices in promoting medication adherence, a randomized controlled study demonstrated that a smartwatch equipped with medication management functions can improve patients’ medication adherence and symptom control (32). Another study that systematically reviewed “wearables and medication adherence” reported that existing evidence supports the potential of wearable devices in enhancing adherence, yet also indicated that integrating wearables into routine clinical practice remains challenging (33).

In terms of multimodal reminders, smartphone screen notifications are the most common approach. One study reported that 64.5% of patients expressed willingness to use mobile text message reminders to maintain medication adherence (34). Regarding voice-based reminders, research has shown that medication reminder functions delivered through voice assistants can help improve adherence (35, 36). For haptic reminders, prior work demonstrated that a low-cost wearable system providing frequent and lightweight haptic cues can effectively motivate older adults undergoing stroke rehabilitation to continue graded exercises, significantly improving their rehabilitation adherence and overall user experience (37).

Therefore, existing evidence provides limited comparative insight into how older adults evaluate common multimodal reminder combinations. Accordingly, we include reminder modality and wearable integration as experimental attributes and compare screen-only, haptic + screen, and voice + haptic configurations.

### 2.3 Environmental micro-adaptation and biophilic design

A growing body of research across multiple domains has shown that incorporating natural elements at various spatial scales, from community green spaces and neighborhood parks to architectural and indoor environments, can positively influence the physiological, cognitive, and emotional states of older adults (38–45). One study reported that indoor environments incorporating biophilic design were found to improve cognitive function and reduce stress among older adults with diabetes (46). In addition, studies on context-aware systems in the living environments of older adults have shown that these systems sense environmental variables such as lighting, noise, and activity levels and adjust feedback or device behaviors accordingly, which can enhance safe living and overall quality of life (47–49). These effects are often attributed to the presence of perceptible restorative cues rather than the physical environment itself, suggesting that environmental signals can be abstracted as designable and adjustable elements in mediated contexts (50–53).

Reflecting this shift, research on digital interaction increasingly explores how micro-level interface environments may support user comfort, emotional regulation, and engagement. However, existing investigations remain limited. First, many remain at the level of conceptual models or small-scale pilot implementations and primarily focus on macro-level planning of architectural or home environments, without incorporating environmental themes as variable systems in interaction interface design experiments (54). Second, empirical research examining how different environmental themes interact with interfaces or wearable devices is still limited, and the role of environmental micro-adaptation in digital health interaction design has not yet been systematically clarified.

Based on these findings, this study incorporates environmental themes as one of the experimental attributes and defines three levels: neutral theme, biophilic theme, and context-adaptive theme. These levels are examined together with other variables, including reminder modality, confirmation method, font and layout, and wearable integration, within the discrete choice experiment. The aim is to investigate the role of environmental micro-adaptation in interface design for medication management among older adults.

### 2.4 Experimental attributes and levels

These five attributes were integrated within a single DCE framework because they jointly shape older adults’ interaction experience in medication-management scenarios. Prior research has shown that reminder cues, interaction burden, typographic accessibility, cross-device coordination, and environmental cues are each associated with users’ attention, effort, or adherence-related behavior, although these effects have typically been examined in isolation or in limited combinations rather than within an integrated framework (25, 27, 32, 49, 51). Examining them together therefore provides a more ecologically valid understanding of how older adults evaluate medication-adherence interfaces as holistic systems.

Based on the literature, five attributes were selected: reminder modality, confirmation method, font and layout, wearable integration, and environmental adaptation. Their corresponding levels and definitions, as well as supporting references, are summarized in Table 1.

**Table 1:** Experimental attributes, levels, and definitions.

Attribute	Levels	Definition and description	References
Reminder modality	Screen	Displays medication reminders in the form of visual pop-up windows, commonly seen in standard app interfaces.	(75, 16, 17, 18, 34)
	Haptic + screen	Provides both vibration and screen feedback through dual channels to reduce missed reminders and enhance response timeliness.	(76, 35, 37, 36, 77)
	Voice + haptic	Combines voice announcements with vibration alerts to support users with reduced visual sensitivity or those in motion.	(35, 36, 78, 79, 77)
Confirmation method	Single tap	Users can confirm medication intake by lightly touching a button, offering simple operation with low cognitive load.	(80, 22, 81)
	QR input	Confirmation is completed via QR code scanning, which ensures security but requires longer task completion time.	(82, 23, 29, 30)
Font and layout	Fixed	Text size and layout remain fixed; the interface is clean but limited in accessibility.	(83, 25, 26)
	Adaptive	Font size automatically adjusts to screen size or viewing distance, improving readability and comfort.	(27, 23, 30, 28)
Wearable integration	Smartphone only	All reminders are displayed on the smartphone, suitable for static usage scenarios.	(80, 75, 24)
	Smartphone + smartwatch	Smartphone and smartwatch reminders are synchronized, allowing users to raise their wrist for quick confirmation and smoother interaction.	(33, 32, 31, 77)
Environmental adaptation	Neutral theme	Uses neutral colors and minimal visual elements, with no additional environmental or contextual cues.	(84, 22, 40, 81)
	Biophilic theme	Incorporates nature-inspired interface elements, such as soft natural color palettes, plant-themed icons, or background imagery, to create a visually calm and pleasant interface atmosphere.	(46, 51, 38, 39, 52, 53, 41, 42, 43)
	Context-adaptive theme	Dynamically adjusts interface visual elements (e.g., background brightness, color tone, or contrast) based on contextual factors such as ambient light, time of day, or usage context to enhance visual comfort and contextual fit.	(50, 51, 48, 85, 49, 53)

In the experimental prototypes, environmental adaptation was operationalized at the interface level rather than through changes to the physical environment. The biophilic theme emphasized the inclusion of nature-inspired visual cues, such as natural color schemes and background imagery, while remaining visually static across contexts. In contrast, the context-adaptive theme focused on dynamic visual adjustments of the interface (e.g., brightness or color tone) in response to contextual factors such as ambient light or time of day. This distinction allowed us to examine whether older adults’ preferences differ between static nature-related cues and context-responsive interface adaptations.

## 3 Materials and methods

### 3.1 Study design and setting

The study employed a discrete choice experiment as the central methodological framework and was conducted in real-world settings with on-site participant recruitment and data collection. The objective was to quantify older users’ preference structures for combinations of interface, wearable, and environmental attributes and to examine the roles these elements played within medication adherence scenarios. This design followed preference modeling approaches commonly used in health interface research (55) and established a closer connection between user preferences and user behavior (56, 57).

### 3.2 Discrete choice experiment design

The discrete choice experiment (DCE) incorporated the five attributes identified in the literature review: reminder modality, confirmation method, font and layout, wearable integration, and environmental adaptation. These attributes were operationalized into experimentally testable levels, as shown in Table 1.

Unlike prior work that focused on modifying a single interface element, this study sought to include multidimensional characteristics of interface interaction when selecting attributes. In particular, two new variables, adaptive font and layout, and environmental micro-adaptation, were incorporated to better capture the layered needs of older adults in real medication scenarios (19, 28, 58).

**Figure 1:**
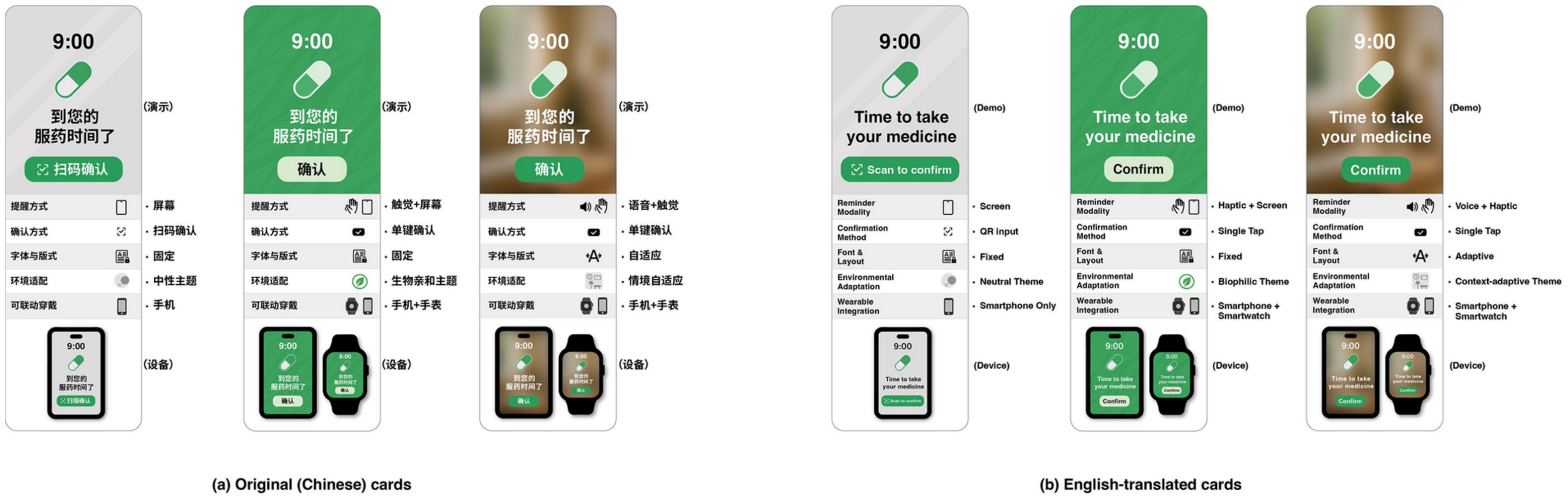
Sample interface images used in the choice experiment **(a)** Original Chinese interface cards and **(b)** the corresponding English-translated versions of the same scenarios. The first scenario (ID 1-2) includes a screen-based reminder, QR input, fixed font and layout, a neutral theme, and smartphone-only integration. The second scenario (ID 8-1) illustrates a haptic + screen reminder with single-tap confirmation, fixed font and layout, a biophilic theme, and smartphone + smartwatch integration. The third scenario (ID 1-1) presents a voice + haptic reminder with single-tap confirmation, adaptive font and layout, a context-adaptive theme, and smartphone + smartwatch integration. These examples were selected from the 24 scenario cards used in the experiment.

To improve experimental efficiency and avoid redundancy, this study adopted a D-efficient design, which reduces the number of choice cards while maintaining the precision of parameter estimation, thereby enabling participants to provide more efficient data contributions within a limited number of tasks (59, 60). Considering that the participants in this study were older adults who primarily use Chinese, the choice cards were presented in Chinese to ensure full comprehension of interface content, reduce cognitive burden, and enhance task engagement. Participants completed 12 choice sets, each containing two alternatives (24 cards in total). This structure supported intuitive understanding of the design schemes and enhanced engagement during the experiment, as shown in Table 2. Figure 1 presents sample interface images illustrating the task scenarios encountered by participants.

**Table 2:** The scenario cards.

ID	Reminder modality	Confirmation method	Font and layout	Wearable integration	Environmental adaptation
1-1	Voice + haptic	Single tap	Adaptive	Smartphone + smartwatch	Context-adaptive theme
1-2	Screen	QR input	Fixed	Smartphone	Neutral theme
2-1	Haptic + screen	Single tap	Adaptive	Smartphone + smartwatch	Biophilic theme
2-2	Screen	Single tap	Fixed	Smartphone	Neutral theme
3-1	Voice + haptic	Single tap	Fixed	Smartphone + smartwatch	Biophilic theme
3-2	Haptic + screen	QR input	Adaptive	Smartphone	Neutral theme
4-1	Haptic + screen	Single tap	Fixed	Smartphone	Context-adaptive theme
4-2	Voice + haptic	QR input	Adaptive	Smartphone + smartwatch	Neutral theme
5-1	Voice + haptic	Single tap	Adaptive	Smartphone	Biophilic theme
5-2	Screen	QR input	Fixed	Smartphone + smartwatch	Context-adaptive theme
6-1	Haptic + screen	Single tap	Adaptive	Smartphone + smartwatch	Neutral theme
6-2	Screen	QR input	Fixed	Smartphone	Biophilic theme
7-1	Voice + haptic	Single tap	Fixed	Smartphone	Context-adaptive theme
7-2	Screen	QR input	Adaptive	Smartphone + smartwatch	Biophilic theme
8-1	Haptic + screen	Single tap	Fixed	Smartphone + smartwatch	Biophilic theme
8-2	Screen	Single tap	Adaptive	Smartphone	Context-adaptive theme
9-1	Voice + haptic	Single tap	Adaptive	Smartphone + smartwatch	Neutral theme
9-2	Haptic + screen	QR input	Fixed	Smartphone	Context-adaptive theme
10-1	Haptic + screen	Single tap	Adaptive	Smartphone	Context-adaptive theme
10-2	Screen	QR input	Fixed	Smartphone + smartwatch	Neutral theme
11-1	Voice + haptic	Single tap	Fixed	Smartphone + smartwatch	Context-adaptive theme
11-2	Screen	Single tap	Adaptive	Smartphone	Neutral theme
12-1	Haptic + screen	Single tap	Adaptive	smartphone	Neutral theme
12-2	Screen	QR input	Fixed	Smartphone + smartwatch	Context-adaptive theme

### 3.3 Participants and procedures

The participants in this study were adults aged 60 years and above who were able to independently operate a smartphone and complete basic interface interactions. Individuals with severe visual or hearing impairments or with moderate to severe cognitive impairment were excluded. The target sample size was 200 participants in order to ensure adequate statistical power for the multi-attribute discrete choice experiment (DCE) (61, 62).

Eligibility was assessed prior to participation through brief screening questions and functional communication checks conducted by trained research team members. Older adults who reported severe visual or hearing impairments, moderate to severe cognitive impairment, or who were unable to understand the task instructions were excluded. In addition, participants were required to be currently taking at least one medication; individuals reporting no medication use at the time of recruitment were not included, as the study focused on medication-adherence decision-making.

Recruitment primarily occurred in high-traffic public locations, including shopping malls, community activity centers, and parks. As a token of appreciation, participants received a small gift (e.g., a pack of tissues or wet wipes) after completing the study tasks.

Research team members randomly invited eligible older adults and, after obtaining informed consent, introduced the study purpose and task instructions using illustrated explanations. After explaining the study content and ensuring that participants fully understood the procedures, the team presented DCE cards containing different interface combinations for participants to make choices based on their personal preferences. All participants included in the final analysis completed all 12 choice tasks. Each participant required approximately 15 min to complete the task. Before the experiment, the research team informed all participants that their data would remain anonymous and would be used solely for academic research.

A total of 253 questionnaires were distributed, and 220 were returned (response rate: 87.0%). Returned questionnaires were excluded from analysis if participants did not complete all choice tasks or left one or more items unanswered, primarily due to time constraints or loss of engagement during the survey. After excluding incomplete or invalid responses, 203 valid samples were obtained, yielding an effective rate of 92.3%. All valid participants met the inclusion criteria described above (see Table 3). Of these participants, 41.4% were male (*n* = 84) and 58.6% were female (*n* = 119).

**Table 3:** Demographic and background characteristics of participants.

Variable	Category	Sample size	Percentage (%)
Gender	Male	84	41.4
	Female	119	58.6
Age group	60–64 years	147	72.4
	65–69 years	35	17.2
	≥70 years	21	10.3
Digital literacy	Low	46	22.7
	Medium	128	63.1
	High	29	14.3
Medication complexity	Single medication	59	29.1
	2–3 medications	122	60.1
	≥4 medications	22	10.8

As shown in Table 3, the sample included a broad distribution across age groups, digital literacy levels, and medication complexity. Most participants were aged 60–64 years, reported medium digital literacy, and were concurrently taking two to three medications. Overall, the sample demonstrated sufficient diversity to support subsequent model estimation and analyses of preference heterogeneity.

### 3.4 Materials and procedure

The experimental materials consisted of high-fidelity digital interface prototypes presented on a tablet device. During the experiment, trained research assistants provided on-site guidance to ensure that participants clearly understood the task requirements and interface content before making their selections. The prototypes were demonstrated directly on the tablet, and participants made preference choices based on what they viewed on the screen, with assistance available when clarification was needed.

Digital literacy was assessed using a self-report checklist consisting of four common technology-related tasks: the ability to use basic functions of mobile applications such as WeChat, the ability to install or uninstall mobile applications, the ability to use a smartwatch or fitness band, and the ability to perform only the most basic operations. Participants could select all options that applied. Based on the number of tasks selected, digital literacy was categorized as low (one task), medium (two tasks), or high (three or four tasks).

Before the formal experiment, the research team conducted a small-scale pilot test to confirm the comprehensibility and operational feasibility of the materials. The experimental procedure included two stages. First, basic information was collected from participants, including gender, age group, digital literacy level, and medication usage. Subsequently, participants completed preference selections for 12 choice sets consisting of 24 scenario cards (55).

### 3.5 Data analysis

Data analysis was conducted at two levels. First, at the level of overall preference estimation, the mixed logit model (MLM) was used to estimate the part-worth utilities and relative importance of the five attribute categories. This model incorporates random coefficients to capture preference heterogeneity across individuals and is well-suited for analyzing choice behaviors involving multidimensional interface attributes (63–66). The utility function can be expressed as follows:


$$U_{\mathrm{ijt}}=\beta_{i}^{\prime }X_{\mathrm{ijt}}+\varepsilon_{\mathrm{ijt}}$$

In this formulation, $U_{\mathrm{ijt}}$ represents the overall utility that individual i assigns to alternative j in choice situation t . The vector $X_{\mathrm{ijt}}$ denotes the attribute levels, $\beta_{i}$ is the individual-specific coefficient vector, and $\varepsilon_{\mathrm{ijt}}$ is the random error term that follows an independently and identically distributed (i.i.d.) structure.

To account for preference differences across individuals, several attribute coefficients were specified as random parameters in this study. In the Mixed Logit specification, the non-reference levels of all five attributes were modeled as random parameters. Specifically, random coefficients were estimated for the following levels: voice + haptic reminder, haptic + screen reminder, QR-input confirmation, adaptive font and layout, smartphone–smartwatch integration, context-adaptive theme, and biophilic theme. The reference levels were treated as fixed to maintain identification.

To further examine preference differences within the older adult population, this study employed latent class analysis (LCA) to segment the sample into several latent groups with distinct preference structures (67–70). The mathematical expression is as follows:


$$P_{\mathrm{ijt}}=\sum_{s=1}^{S}\pi_{s}\frac{\exp(\beta_{s}^{\prime }X_{\mathrm{ijt}})}{\sum_{k=1}^{J}\exp(\beta_{s}^{\prime }X_{\mathrm{ikt}})}$$

In this expression, S denotes the number of latent classes, $\pi_{s}$ represents the prior probability of belonging to class s with the constraint that $\sum_{s=1}^{S}\pi_{s}=1$ , and $\beta_{s}$ represents the vector of attribute coefficients corresponding to class s . The denominator sums over all alternatives k in choice situation t . Model fit was compared using the Akaike Information Criterion (AIC) and the Bayesian Information Criterion (BIC) to determine the optimal number of classes.

All statistical analyses were conducted in StataMP 18.0 using the mixlogit and lclogit commands, and statistical significance was set at *p* < 0.05.

## 4 Results

### 4.1 Estimation of attribute utilities

Based on the mixed logit model (MLM), utility values were estimated for all five attributes (Table 4). For the reminder modality, both “voice + haptic” (*β* = 0.671, *p* < 0.001) and “haptic + screen” (*β* = 0.265, *p* < 0.01) were significantly preferred over screen-only reminders, indicating a strong inclination among older adults toward multimodal prompting. For the confirmation method, “QR input” showed a negative and highly significant effect (*β* = −0.811, *p* < 0.001), suggesting a clear preference for the lower-burden “single-tap” confirmation. Regarding typography, the adaptive layout option yielded a positive utility estimate (*β* = 0.283, *p* < 0.001), underscoring the importance of dynamic font adjustments in reducing visual strain. For device integration, “smartphone + smartwatch” also generated a significant positive effect (*β* = 0.287, *p* < 0.001), reflecting a preference for coordinated cross-device interactions. Finally, both environmental themes showed substantial positive utilities, context-adaptive theme (*β* = 0.771, *p* < 0.001) and biophilic theme (*β* = 0.605, *p* < 0.001), with the former producing the highest utility estimate among all attribute levels, suggesting that contextual alignment in visual themes may enhance motivation and engagement.

**Table 4:** Estimated coefficients for attribute levels.

Attributes (base level)	Levels	Coefficient (*β*)	SD parameter
Reminder modality (base = screen)	Haptic + screen	0.265**	0.240
	Voice + haptic	0.671***	−0.007
Confirmation method (base = single tap)	QR input	−0.811***	0.482***
Font and layout (base = fixed)	Adaptive	0.283***	0.010
Wearable integration (base = smartphone)	Smartphone + smartwatch	0.287***	0.173
Environmental adaptation (base = neutral theme)	Context-adaptive theme	0.771***	0.354**
	Biophilic theme	0.605***	0.105

*p < 0.05; **p < 0.01; ***p < 0.001. “SD Parameter” represents the estimated coefficient of the random-parameter standard deviation in Stata’s mixlogit output. Its sign has no substantive interpretation; the implied standard deviation is positive.

### 4.2 Group heterogeneity

To further identify the optimal classification of preference differences among older adults, this study conducted model fit comparisons using latent class analysis (LCA). Among the two- to five-class models tested, the two-class model provided the best overall fit, showing the lowest BIC and an AIC value that was highly comparable to the more complex specifications (Table 5). Therefore, it was selected as the optimal solution for representing preference heterogeneity (71, 72).

**Table 5:** Model fit statistics for latent class models.

Number of classes	AIC	BIC
2-class model	2,664.681	2,807.489
3-class model	2,658.586	2,898.763
4-class model	2,651.146	2,969.218
5-class model	2,653.206	3,068.647

**Figure 2:**
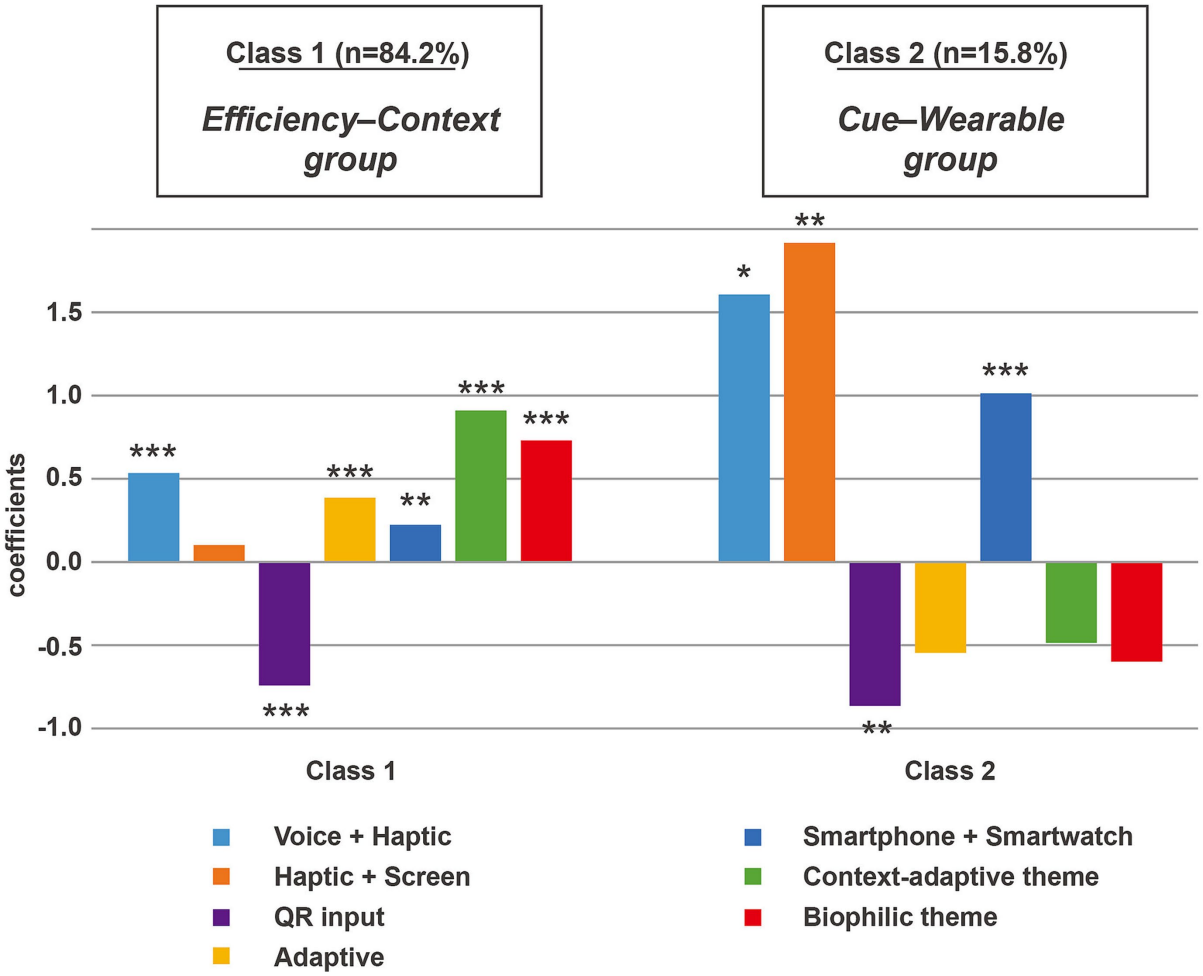
Latent class analysis: attribute-level coefficients by class.

Therefore, this study adopted the two-class model as the optimal latent class solution (Table 6) and reported the attribute-level coefficients for each class (Figure 2). The class proportions were 84.2% and 15.8%, respectively. Class 1, characterized as the Function-Efficiency and Contextual-Adaptation group (hereafter referred to as the Efficiency–Context group), showed significant preferences for voice + haptic reminders, adaptive font and layout, smartphone + smartwatch, and both context-adaptive and biophilic themes, while strongly rejecting QR input. The haptic + screen reminder was not significant in this class. These results indicate that most participants preferred lower operational burden and clear multimodal feedback and supported contextual adaptation in layout and environmental themes.

**Table 6:** Latent class analysis results.

Attributes (base level)	Levels	Class 1		Class 2	
		Coefficient	SE	Coefficient	SE
Reminder modality (base = screen)	Voice + haptic	0.528***	(0.121)	1.602*	(0.659)
	Haptic + screen	0.096	(0.108)	1.911**	(0.652)
Confirmation method (base = single tap)	QR input	−0.746***	(0.090)	−0.870**	(0.314)
Font and layout (base = fixed)	Adaptive	0.379***	(0.082)	−0.552	(0.453)
Wearable integration (base = smartphone)	Smartphone + smartwatch	0.215**	(0.062)	1.006***	(0.250)
Environmental adaptation (base = neutral theme)	Context-adaptive theme	0.905***	(0.087)	−0.491	(0.294)
	Biophilic theme	0.726***	(0.101)	−0.604	(0.361)
Class proportion	—	0.842		0.158	
Class membership model parameters					
Male		−1.673*		0	
60–64 years		−0.045		0	
65–69 years		−3.801		0	
Low digital literacy		1.023		0	
Medium digital literacy		4.494		0	
Single medication		5.517		0	
2–3 medications		1.310		0	
Constant		−0.403		0	

SE = Standard error. *p < 0.05; **p < 0.01; ***p < 0.001.

Class 2 can be characterized as the Cue-Dominant and Wearable-Integration group (hereafter referred to as the Cue–Wearable group), which showed significant preferences for voice + haptic reminders, haptic + screen reminders, and smartphone + smartwatch, and likewise demonstrated a strong aversion to QR input. This class showed only weak or nonsignificant preferences for adaptive font and layout, and displayed negative but nonsignificant estimates for the context-adaptive and biophilic themes. This group placed greater emphasis on the strength of reminders and the accessibility facilitated by device coordination, and was not particularly sensitive to layout adaptivity or environmental themes. Taken together, Class 1 placed emphasis on reducing interaction burden and supporting contextual adaptivity, whereas Class 2 prioritized strong reminder cues and device coordination while showing limited sensitivity to layout or environmental themes. Both classes exhibited a strong aversion to QR input, indicating a consistent need among participants to minimize operational steps and reduce interaction burden.

In the class membership model, gender showed a significant effect on class assignment. Specifically, the coefficient for males was negative (*β* = −1.673, *p* < 0.05), indicating that men were significantly less likely to belong to Class 1. Conversely, women were more likely to fall into the Function–Efficiency group, which may suggest greater adaptability in information acquisition and interface use. Although age, digital literacy level, and medication complexity showed directional trends in the model, none reached statistical significance, indicating that the influence of these variables on class assignment remained unstable given the sample size.

## 5 Discussion

### 5.1 Main findings

Based on the results of the discrete choice experiment, this study identified the core interface preferences of older adults in medication adherence scenarios and revealed meaningful group differences. Overall, older users showed a clear preference for interface combinations that include multimodal reminders, adaptive font and layout, low-burden confirmation methods, and cross-device coordination. At the same time, alignment between interface features and environmental characteristics significantly enhanced willingness to interact and adherence-related behavior.

### 5.2 Comparison with prior work

First, regarding reminder modality, the combination of voice and haptic cues was preferred over visual-only reminders. This finding is consistent with previous research indicating that multisensory input can enhance adherence and safety (34–37). However, our results suggest that participants preferred multimodal coordination over single-modality reminders, a pattern that may translate into more effective prompting in real-world use. This may be explained by multisensory integration mechanisms, as combining auditory and haptic cues produces redundancy gains that improve detectability and reduce missed reminders, particularly for older adults who often experience declining visual and attentional resources (73, 74).

In addition, QR input was strongly rejected, indicating that complex interaction procedures reduce willingness to use the interface among older users. This contrasts with earlier approaches that emphasized QR-based verification for ensuring safety and accuracy (29, 30), and highlights the need for designers to pay closer attention to the negative effects of operational burden and attentional shifts in older user scenarios. For medication-related applications, this suggests that confirmation mechanisms should prioritize low-burden interactions—such as single-tap confirmation or automated logging—rather than relying on QR-based workflows that interrupt task flow and impose unnecessary motor and attentional demands.

Regarding font and layout, this study found that the adaptive layout produced a significant positive utility. This finding not only supports previous research showing that font readability affects interface friendliness for older adults (25–27), but also further suggests that dynamic adaptation is more aligned with the visual and cognitive needs of older users than conventional static optimization (28). In other words, interfaces that automatically adjust font size, letter spacing, and contrast based on contextual conditions offer a better reading experience than simply enlarging text within a fixed layout.

In terms of device coordination, the combination of smartphone + smartwatch was strongly preferred, indicating that cross-device consistency can effectively reduce the cognitive cost associated with context switching. Unlike studies that advocate for the superiority of a single wearable device (31–33), our findings indicate that older adults expressed a clear preference for coordinated rather than substitutive device use. When smartphones and smartwatches maintain synchronized information and consistent interaction logic, this preference may reflect the reduced cognitive effort associated with smoother transitions across devices.

Regarding environmental factors, both the context-adaptive and biophilic themes produced positive utilities. While prior studies have primarily examined physical environmental conditions such as lighting and temperature (46, 48, 49, 54), these investigations highlight the broader role of environmental cues in shaping users’ affective and cognitive states. Extending this perspective, our findings suggest that micro-level environmental themes embedded in the interface may influence users’ willingness to engage by functioning as perceived restorative cues, rather than by reproducing physical environmental conditions, thereby supporting emotional comfort and visual clarity (51, 53).

### 5.3 Implications for design

The latent class analysis revealed heterogeneity in user preferences. Most older adults belonged to the Efficiency–Context group, which emphasized simplicity of operation and contextual alignment, whereas another subset of users belonged to the Cue–Wearable group, which placed greater emphasis on reminder strength and device coordination. The former sought a lightweight and stable experience, while the latter required more salient prompting functions. These patterns contribute to the theoretical understanding of heterogeneity in aging-related interaction behaviors, suggesting that older adults do not form a homogeneous group but instead organize their preferences around distinct cognitive and perceptual priorities. These findings extend previous research that has primarily examined older users at an aggregate level or along limited design dimensions, and demonstrate that within-group preference differences should not be overlooked (26, 27, 55). It is worth noting that, apart from gender, other demographic variables did not show statistically significant associations with class membership, and thus subgroup differences should be interpreted as preference patterns rather than demographic divisions. From an application design perspective, this implies that medication-adherence tools may benefit from adaptive or personalized interface pathways, offering streamlined, low-burden interactions for one group and more salient cueing and cross-device coordination for the other. Future interface design should therefore incorporate stratified optimization tailored to different user types.

Taken together, the findings provide a systematic response to the research questions proposed earlier regarding older adults’ interface preferences and preference heterogeneity in medication-adherence scenarios. The results indicate that older adults exhibit clear multidimensional preferences and notable group differences in the design of medication-adherence interfaces. The discrete choice experiment revealed how older users evaluate and prioritize combinations of reminder modality, confirmation burden, font and layout, wearable integration, and environmental adaptation within medication-management scenarios. In addition, the latent class analysis demonstrated that these preferences are not homogeneous, but instead cluster into distinct patterns characterized by different emphases on operational efficiency, contextual alignment, reminder salience, and device coordination. These results highlight the importance of considering both overall preference structures and user heterogeneity when designing age-friendly medication-adherence systems.

### 5.4 Limitations and future research

This study has several limitations. First, the scenario-based tasks and short-term preference choices may not fully reflect long-term, real-world medication-adherence behaviors. Second, the sample mainly consisted of older adults with basic levels of digital literacy, and the generalizability of the findings to individuals with lower literacy or more severe cognitive impairments requires further investigation. Third, the study did not examine specific interaction parameters, such as tactile intensity, voice characteristics, or detailed typographic settings, which may influence user experience during actual use.

Future research should therefore focus on longitudinal studies conducted in real-world contexts to validate the relationship between stated preferences and observed behaviors, as well as on parameterized experiments exploring optimal combinations of reminder modalities, typographic configurations, and environmental adaptation mechanisms. In addition, incorporating factors such as psychological state, emotional responses, and cognitive load may help develop a more comprehensive interaction model for older adults.

## 6 Conclusion

This study employed a discrete choice experiment to examine older adults’ preferences for medication-adherence interface design across multiple attributes, including reminder modality, confirmation method, font and layout, wearable integration, and environmental adaptation. The results demonstrate clear multidimensional preference structures as well as notable heterogeneity among older users, with distinct groups emphasizing operational efficiency, contextual alignment, reminder salience, and device coordination. These findings highlight the importance of considering both overall preference patterns and user heterogeneity when designing age-friendly digital health interfaces and provide a quantitative foundation for future research and design efforts in medication-adherence systems.

## Data Availability

The original contributions presented in the study are included in the article/supplementary material, further inquiries can be directed to the corresponding author.
